# Remarkable regression of massive deep vein thrombosis in response to intensive oral rivaroxaban treatment

**DOI:** 10.1186/s12959-015-0045-1

**Published:** 2015-03-14

**Authors:** Norimichi Koitabashi, Nogiku Niwamae, Tetsuya Taguchi, Yoshiaki Ohyama, Noriaki Takama, Masahiko Kurabayashi

**Affiliations:** Department of Medicine and Biological Sciences, Gunma University Graduate School of Medicine, 3-39-22, Showa-machi, Maebashi, Gunma 371-8511 Japan; Department of Cardiovascular Medicine, Maebashi Red Cross Hospital, Maebashi, Gunma Japan; Department of Internal Medicine, Fukaya Red Cross Hospital, Fukaya, Saitama Japan

**Keywords:** Rivaroxaban, Deep vein thrombosis, Anticoagulation, Iliac vein compression, Thrombus regression

## Abstract

Deep vein thrombosis (DVT) is a common disease and is associated with pulmonary embolism (PE). Proximal iliofemoral DVT may lead to severe PE and chronic venous insufficiency. The standard therapy for DVT is anticoagulant therapy using heparin and a vitamin K antagonist, but a recent clinical study showed that rivaroxaban, an oral Xa inhibitor, was comparable to standard therapy and had less bleeding complications. Intensive high-dose anticoagulation is recommended during the initial 3 weeks of DVT treatment. The present report describes a case of a 77-year-old male showing a remarkable regression of DVT in response to rivaroxaban treatment within the initial 3 weeks of therapy and who did not experience any adverse events. His DVT was massive and was accompanied by proximal iliofemoral vein thrombus and iliac vein compression syndrome. Rivaroxaban, especially in intensive high-dose treatment, might be a safe and effective therapeutic choice for massive DVT.

## Background

Venous thromboembolism (VTE), including deep vein thrombosis (DVT) and pulmonary embolism (PE), is a common medical condition and is the third leading cause of cardiovascular death [1]. The clinical severity of DVT is associated with the burden and location of the venous thrombus. Patients with proximal iliofemoral DVT are most likely to have early and late morbidity [2]. Anticoagulant therapy is effective for the management of acute VTE and for the prevention of recurrent events and death associated with recurrent VTE [1,3]. For half a century, the standard therapy for patients with VTE has been the administration of heparin, overlapped and followed by a vitamin K antagonist (VKA) [1]. This regimen is effective but has several limitations, such as requirement for laboratory monitoring and dose adjustments, a narrow therapeutic window, and interaction with other drugs and foods [1,4]. Recently, new direct oral anticoagulants (DOACs), such as dabigatran, rivaroxaban, apixaban and edoxaban, which were developed for stroke prevention in the context of atrial fibrillation [5], have been shown to be effective for the treatment of VTE [6,7]. Rivaroxaban offers a simple, single-drug approach for initial intensive treatment, with high-dose rivaroxaban administered for 3 weeks followed by administration of standard dose rivaroxaban [8]. In the EINSTEIN-DVT [9] and EINSTEIN PE [4], this regimen showed no inferiority and no increase in adverse events when compared with standard therapy (low-molecular weight heparin [LMWH] with VKA).

Following these results, the J-EINSTEIN-DVT and -PE studies have been conducted in Japan [10]. These studies are open-label, randomized, multicenter trials that compared oral rivaroxaban alone with standard therapy in Japanese patients with DVT or PE. We participated in these studies and enrolled several patients. One patient with massive proximal DVT was assigned to rivaroxaban 15 mg twice-daily and showed marked improvement during the first 3 weeks of therapy without any adverse effects including minor bleeding.

## Case presentation

**Figure 1:**
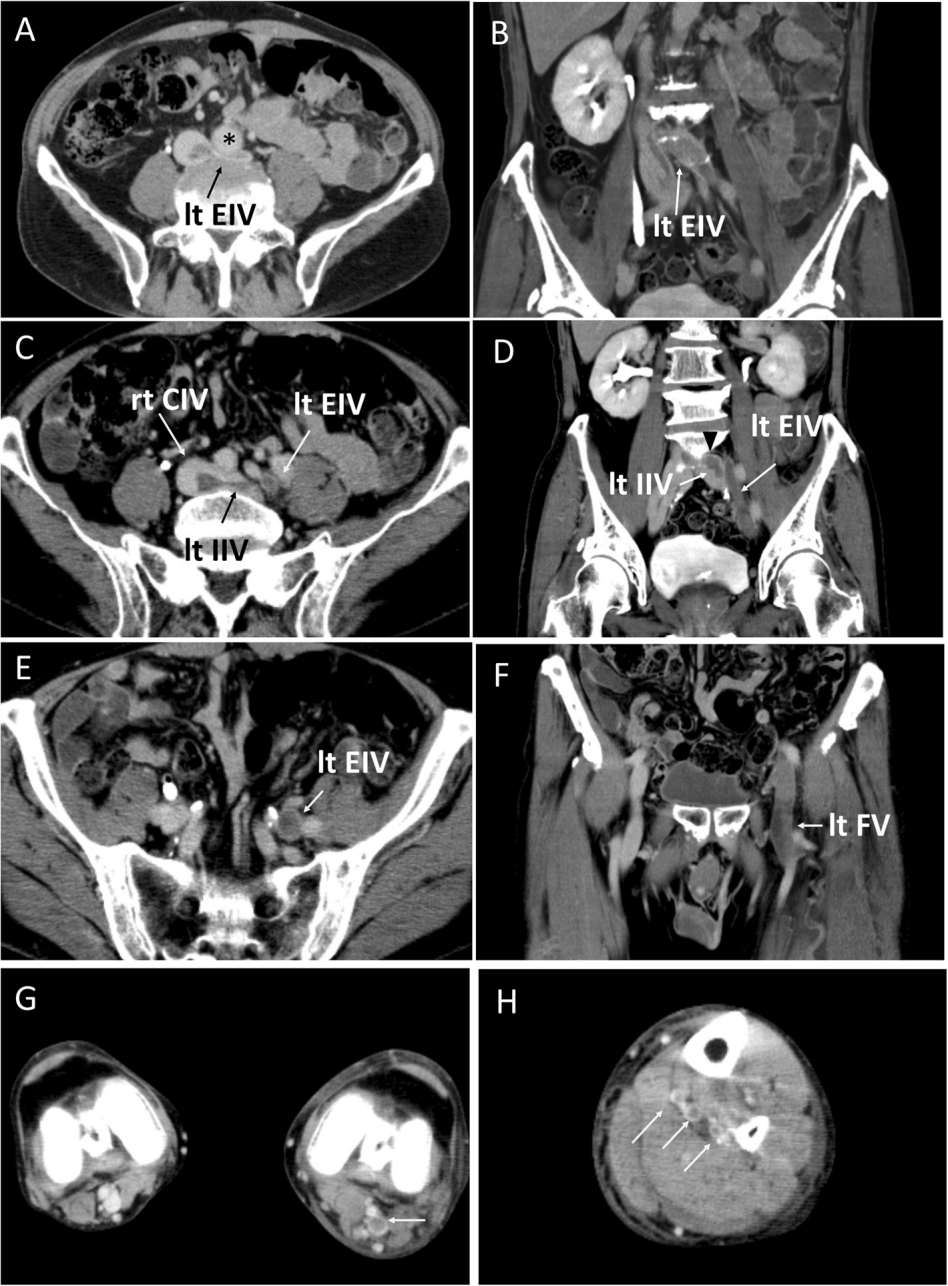
**Multi-detector computed tomography (MDCT) on admission. A** and **B**. Transverse plane **(A)** and coronal plane **(B)** at the ilio-cava junction level. Left external iliac vein (lt EIV) branches off directly from the inferior vena cava. The left EIA is compressed by the abdominal aorta (*) with thrombus; **C** to **E**. Transverse plane **(C and E)** and coronal plane **(D and F)** at the iliofemoral vein level. Lt EIV, right common iliac vein (rt CIV), left internal iliac vein (lt IIV) and left femoral vein (let FV) have thrombi; **G**. left popliteal vein thrombus (white arrow); **H**. thrombi of left lower leg veins/soleal veins (white arrows).

**Figure 2:**
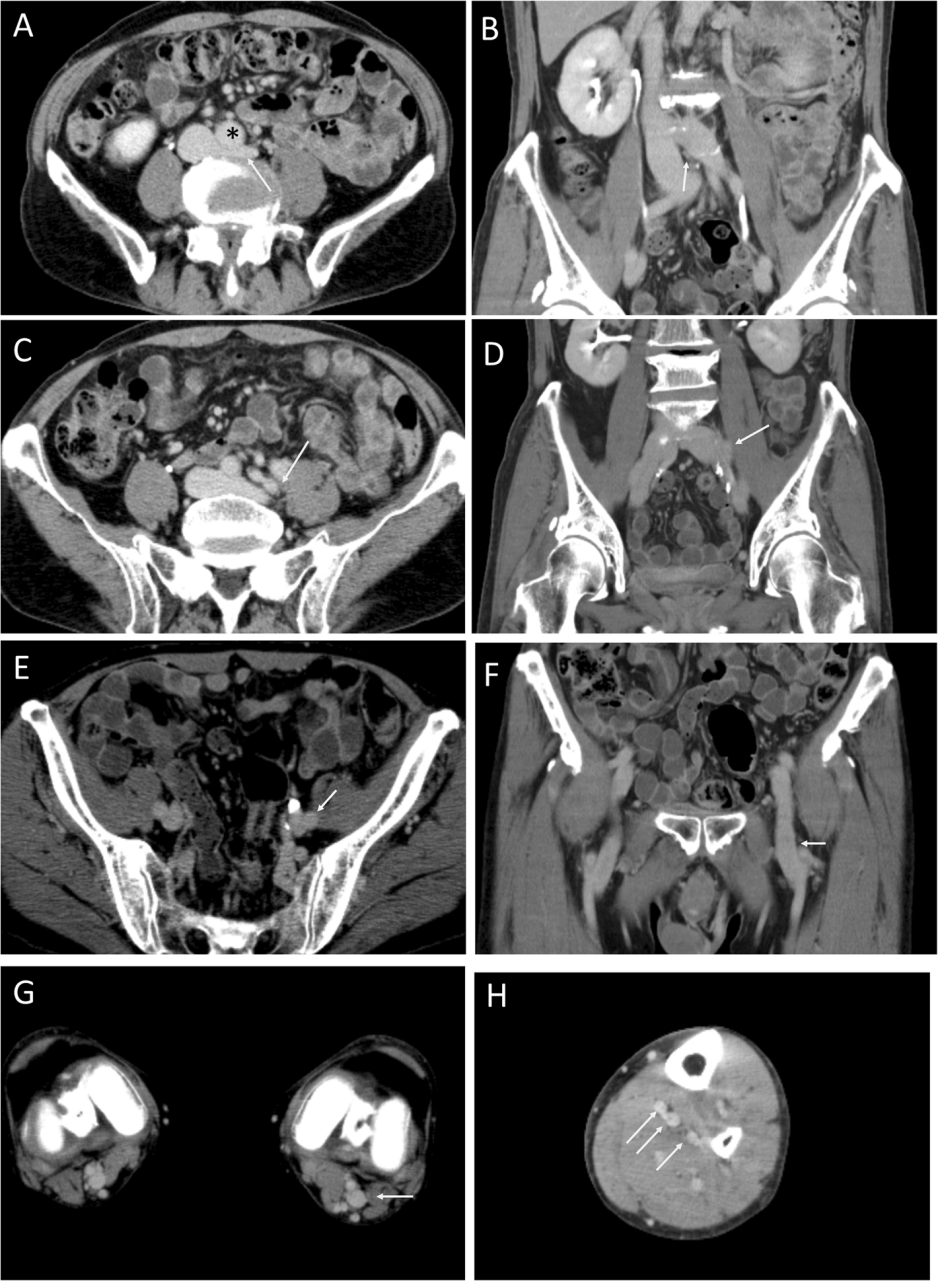
**MDCT at day 22. A** and **B**. Transverse plane **(A)** and coronal plane **(B)** at the ilio-cava junction level. * Abdominal aorta. Iliac vein compression persists, but the contrast defect has disappeared (white arrow); **C** to **E**. Transverse plane **(C and E)** and coronal plane **(D and F)** at the iliofemoral vein level. Thrombi shown in Figure 1 have disappeared (white arrow); **G**. Absence of the left popliteal vein thrombus (white arrow); **H**. Absence of the left lower leg vein thrombus (white arrows).

A 77-year-old male was admitted to our hospital due to left lower limb swelling with heat sensation and redness. These symptoms had appeared 2 days prior to hospital admission. Five months before admission, he was diagnosed with polymyalgia rheumatica syndrome and started treatment with prednisolone 10 mg per day. He had no previous history of thrombosis and no family history suggestive of inherited thrombophilia. His major risk for VTE was advanced age, but he did not have any other risks, such as obesity, cancer, surgery, immobilization or recent long travel. At the time of arrival to our Emergency Department, his entire left leg was swollen and showed pitting edema with mild pain. Blood pressure was 164/78 mmHg, and pulse of 102 bpm. Respiratory rate was 12/min, and arterial oxyhemoglobin saturation was 96% at room air. Plasma D-dimer level was high (45.0 μg/mL) while serum protein level, renal function and liver function were normal. Multi-detector computed tomography (MDCT) showed massive deep vein thrombosis from the lower edge of inferior vena cava to the lower leg veins (Figure 1). This patient had a congenital anomaly of the iliac vein in which the left external iliac vein (EIV) and left internal iliac vein (IIV) separately branched off from the inferior vena cava without a left common iliac vein (Figure 1A). The left EIV was compressed by the abdominal aorta and the fifth lumbar vertebra (Figure 1A and B). The thrombosis extended from the calf veins to the compressed site, suggesting a variant of iliac vein compression syndrome (May-Thurner syndrome) [11,12]. The left IIV also had a large thrombus (Figure 1C and D). The patient did not have any symptom related with PE and his lung MDCT did not show any embolism in the pulmonary arteries. Compression venous ultrasonography also showed massive DVT from the left iliofemoral level to the lower leg level, but slight venous flow was observed by color Doppler at the level of the left common femoral vein. A diagnosis of DVT was made, and the patient was immediately started on intravenous unfractionated heparin administration. The patient subsequently provided written informed consent to participate in the J-EINSTEIN study [10]. We screened thrombophilia including antiphospholipid syndrome, protein S/C deficiency, fibrinogen and antithrombin III abnormality, but could not find any cause of DVT except steroid administration. Then, prednisolone dose was reduced to 7.5 mg. Since the patient was assigned to begin rivaroxaban therapy on the second day of hospitalization, a 15-mg dose of rivaroxaban administration was given 4 hours after termination of heparin, according to the study protocol. His left leg swelling showed progressive reduction on a daily basis in response to rivaroxaban 15 mg twice-daily. After 2 weeks of treatment, the patient was discharged from our hospital, as his symptoms and leg swelling had markedly improved. We did not use any compression therapy because the symptom improvement was observed from initial several days. The circumference of the left femoral region had decreased from 50.6 cm to 45.8 cm over the 2 weeks of initial treatment, and plasma D-dimer level decreased to 3.9 μg/mL by the time of hospital discharge. Surprisingly, the massive DVT in his left leg had almost completely disappeared according to MDCT performed on day 22 of the treatment (Figure 2). Compression venous ultrasonography at day 25 only showed a small thrombus at the left popliteal vein. As per protocol, we switched the dose of rivaroxaban to 15 mg per day following 3 weeks of intensive therapy. We monitored his clinical symptom and physical examination every month and continued the study drug for 1 year. Neither bleeding nor recurrence of thrombosis occurred over the entire clinical course of the treatment.

## Discussion

The present report described a case of a patient with DVT who showed marked improvement in response to oral rivaroxaban treatment during the initial intensive dose period. This patient had a massive thrombus extending from the left iliac vein to the lower leg veins with iliac vein compression syndrome. He had typical symptoms of DVT for 2 days but had no obvious risk factors for acute DVT except for steroid administration. He was enrolled in the J-EINSTEIN study and was assigned to receive rivaroxaban 15 mg twice daily. After starting treatment, his leg edema progressive decreased on a daily basis. CT scan on day 22 revealed that the iliac vein thrombosis had almost completely disappeared. No bleeding event occurred during the course of treatment.

Iliac vein compression syndrome (May-Thurner syndrome) is most commonly seen in women at 30 to 50 years of age [12]. The right common iliac artery crosses over the left common iliac vein and then runs adjacent to the right iliac vein. Typically, the left iliac vein is compressed between the right common iliac artery anteriorly and the sacral promontory or the fifth lumbar vertebra posteriorly just before the iliocaval junction [12]. This case did not have a left common iliac vein and had venous return directly from the left external iliac vein to the vena cava at an unusually proximal position. We assume that this anomaly contributed to the development of iliac compression by the abdominal aorta. Aging-related atherosclerosis and vertebra deformity might anatomically augment the vein compression in this anomaly.

Proximal iliofemoral DVT causes PE in about half of cases. This condition can also lead to chronic venous insufficiency and recurrent thrombosis [2]. Compression of the iliac vein has been documented in about 50% of patients with left iliac DVT [12] and may require treatment via invasive endovascular therapy, such as percutaneous balloon venoplasty and stent implantation [12,13]. However, some degree of compression of the left common iliac vein may also be an incidental finding [14]. Although our case was affected by the iliac vein compression, the decrease in thrombus size in response to medical therapy without the need for invasive approaches suggests that iliac vein compression did not have a significant effect on the response to medical therapy. After 3 weeks of high-dose treatment, the rivaroxaban dose was decreased to complete a 1-year course. The patient did not experience DVT recurrence despite persistent iliac compression.

Rivaroxaban is an oral Xa inhibitor that is as effective as standard anticoagulant therapy (i.e., heparin and warfarin) for VTE treatment [4,9]. Earlier studies showed that an intensive regimen (15 mg twice-daily) during the first 3 weeks is necessary to achieve an effective higher trough levels and earlier steady state and thereby contribute to more effective regression of thrombosis [8]. The J-EINSTEIN study was designed to examine the effectiveness of rivaroxaban for Japanese patients with VTE [10]. A 30-mg dose per day is two times higher than the dose of standard anticoagulant therapy (15 mg per day) for atrial fibrillation in Japan. This high-dose intensive regimen, however, did not cause any significant adverse bleeding events, even in Japanese patients [10].

An important point in VTE treatment is to consider the risk and benefit of anticoagulant therapy, but the effectiveness of the selected therapy for thrombus regression should be considered as well [2,3,15]. Long-standing DVT related to insufficient anticoagulation might increase the risk of postthrombosis syndrome and organization of the thrombus [2,15]. Thrombolysis therapy is associated with a substantial effect on thrombus regression in massive DVT [2,15], while bleeding risk is definitely increased in general [16,17]. Since the imaging technique for quantifying thrombus is limited, there was no comparable data for anticoagulant therapy in terms of ability for thrombus regression. Generally, standard anticoagulant therapy is not quite effective for thrombus regression when compared with thrombolysis therapy [18,19]. Although distal DVT, including those at the femoral level, may spontaneously regress with only anticoagulant therapy [19], the actual effect of the anticoagulant on DVT regression at the level of the iliac vein remains unclear. Surprisingly, the patient in the present case experienced nearly 100% thrombus regression over the first 3 weeks of therapy and did not experience recurrence, implying that the high-dose regimen might be better treatment than standard therapy.

In J-EINSTEIN study, regression of thrombus was more frequently observed in rivaroxaban-treated group compared with heparin-warfarin-treated group [10]. Khalafallah et al. showed a Caucasian case with a remarkable regression of bilateral extensive DVT by rivaroxaban suggesting that the efficacy would not be unique for Japanese patients [20]. However, since follow-up CT scan within a month from initial DVT treatment is generally uncommon in Western countries, there were few reports to show the early phase DVT regression induced by anticoagulant without thrombolysis. It would be worth to compare rivaroxaban and standard anticoagulants in terms of the thrombus-regressive effect in large international cohort in future.

There are several possible reasons for the marked effect of rivaroxaban in the present case. One reason is that the treatment was started during the early phase of DVT [3]. Another reason might be spontaneous recanalization [19], as compression venous ultrasonography showed slight venous flow even before treatment. Thrombus regression in response to anticoagulant therapy is more difficult to achieve in the context of an occluding thrombus. Recent several reports in left atrium or portal vein thrombosis indicate that rivaroxaban might be able to resolve clots rapidly [21-23]. A basic study showed that rivaroxaban-mediated suppression for thrombin generation leads a looser clot which is more degradable by fibrinolytic enzymes [24]. This mechanism may explain its efficacy in promoting the dissolution of the thrombus.

At present, the results of the trials using DOACs in the treatment of VTE showed that these agents are non-inferior and possibly safer than the standard heparin-warfarin regimen [3]. However, in several specific conditions such as cancer, pregnancy and renal insufficiency, the DOAC-use for VTE patents is limited [3]. Experience with DOACs in large patient populations is lacking so far and real-world patient outcome will need to be carefully monitored.

## Conclusions

In summary, this report showed remarkable efficacy and safety of initial intensive treatment with rivaroxaban in a patient with massive thrombus. Further, no bleeding side effects were noted. While large-scale and precise investigation comparing standard treatment and new oral anticoagulants are required to validate these observations, this case suggests that rivaroxaban has the potential to replace conventional therapy with heparin and oral VKA for patients with massive DVT.

## Consent

Written informed consent was obtained from the patient for publication of this Case report and any accompanying images.

## Abbreviations

DOACs: Direct oral anticoagulants
DVT: Deep vein thrombosis
PE: Pulmonary embolism
VTE: Venous thromboembolism
MDCT: Multi-detector computed tomography
EIV: External iliac vein
IIV: Internal iliac vein
CIV: Common iliac vein
